# Supplementary material for: Switch-like Arp2/3 activation upon WASP and WIP recruitment to an apparent threshold level by multivalent linker proteins in vivo
Source: eLife. 2017 Aug 16;6:e29140. doi: 10.7554/eLife.29140 (PMC5559269; doi:10.7554/eLife.29140)
Supplement: Supplementary file 1. — DOI: http://dx.doi.org/10.7554/eLife.29140.030 [file elife-29140-supp1.docx]

Supplemental Table 1

| YSY3171 | *MATa ura3-52 leu2-3,112 his3Δ200 pan1∆PRD-GFP::HIS* |
| --- | --- |
|  |  |
| YSY2701 | *MATa ura3-52 leu2-3,112 his3Δ200 sla1∆::cgLEU, sla1W41AW108AW391A::URA* |
|  |  |
| YSY3291 | *MATa ura3-52 leu2-3,112 his3Δ200 pan1∆PRD-GFP::HIS, sla1∆::cgLEU, sla1 W41AW108AW391A::URA* |
|  |  |
| YSY3414 | *MATa ura3-52 leu2-3,112 his3Δ200 sla1W41AW108A::URA, sla1∆::NatMX* |
|  |  |
| YSY3386 | *MATa ura3-52 leu2-3,112 his3Δ200 pan1∆PRD-GFP::HIS, sla1∆::NatMX, sla1W41AW108A::URA* |
|  |  |
| YSY3413 | *MATa ura3-52 leu2-3,112 his3Δ200 sla1W391A::URA, sla1∆::NatMX* |
|  |  |
| YSY3887 | *MATa ura3-52 leu2-3,112 his3Δ200 pan1∆PRD-GFP::HIS, sla1∆::NatMX, sla1W391A::URA* |
|  |  |
| YSY3416 | *MATa ura3-52 leu2-3,112 his3Δ200 sla1W41AW108A-GFP::URA::KanMX, sla1∆::NatMX* |
|  |  |
| YSY3577 | *MATa ura3-52 leu2-3,112 his3Δ200 myo5CA∆-GFP::KanMX, myo3∆::cgLEU* |
|  |  |
| YSY3578 | *MATa ura3-52 leu2-3,112 his3Δ200 myo5CA∆-GFP::KanMX, myo3∆::cgLEU, sla1∆::NatMX* |
|  |  |
| YSY2678 | *MATa ura3-52 leu2-3,112 his3Δ200 PAN1-mCherry::KanMX, SLA-GFP:HIS* |
|  |  |
| YSY3364 | *MATa ura3-52 leu2-3,112 his3Δ200 pan1∆PRD-mCherry::KanMX, sla1∆::cgLEU, SLA1-GFP::URA::KanMX* |
|  |  |
| YSY3433 | *MATa ura3-52 leu2-3,112 his3Δ200 sla1W41AW108A-GFP::URA::KanMX, sla1∆::NatMX, PAN1-mCherry* |
|  |  |
| YSY3728 | *sla1W41AW108A-GFP::URA::KanMX, sla1∆::NatR* crossed with *pan1∆PRD-mCherry::KanMX* |
|  |  |
| YSY3424 | *MATa ura3-52 leu2-3,112 his3Δ200 SLA2-TagRFP-T::URA, pan1∆PRD-GFP::HIS, sla1W41AW108A::URA, sla1∆::NatMX* |
|  |  |
| YSY3754 | *MATa ura3-52 leu2-3,112 his3Δ200 LAS17-GFP::HIS, pan1∆PRD-13MYC::KanMX, SLA1-mCherry::HIS* |
|  |  |
| YSY3551 | *MATa ura3-52 leu2-3,112 his3Δ200 sla1W41AW108A-mCherry::KanMX::URA, sla1∆::NatMX, LAS17-GFP::HygMX* |
|  |  |
| YSY3529 | *MATa ura3-52 leu2-3,112 his3Δ200 sla1W41AW108A::URA, sla1∆::NatMX, pan1∆PRD-13MYC::KanMX, LAS17-GFP::HIS, ABP1-RFP::HygMX* |
|  |  |
| YSY3418 | *MATa ura3-52 leu2-3,112 his3Δ200 SLA1-GFP::URA::KanMX, sla1∆::cgLEU, ABP1-RFP::HygMX* |
|  |  |
| YSY3397 | *MATa ura3-52 leu2-3,112 his3Δ200 pan1∆PRD -13MYC::KanMX, sla1∆::cgLEU, SLA1-GFP::URA::KanMX, ABP1-RFP::HIS* |
|  |  |
| YSY3432 | *MATa ura3-52 leu2-3,112 his3Δ200 sla1W41AW108A-GFP::URA::KanMX, sla1∆::NatMX, ABP1-RFP::HygMX* |
|  |  |
| YSY3478 | *MATa ura3-52 leu2-3,112 his3Δ200 sla1∆::NatMX, sla1W41AW108A::URA, pan1∆PRD-13MYC::KanMX, SAC6-GFP, PRD5-RFP::HIS* |
|  |  |
| YSY3561 | *YSY3416* crossed with *YSY3466* |
|  |  |
| YSY3466 | *MATa ura3-52 leu2-3,112 his3Δ200 pan1∆PRD-13MYC::KanMX, ABP1-RFP::HygMX* |
|  |  |
| YSY3528 | *MATa ura3-52 leu2-3,112 his3Δ200 sla1W41AW108A::URA, sla1∆::NatMX, pan1∆PRD-13MYC::KanMX, LAS17-GFP::HIS, ABP1-RFP::HygMX* |
|  |  |
| YSY3500 | *MATa ura3-52 leu2-3,112 his3Δ200 VRP1-GFP::HIS, ABP1-RFP::HygMX, pan1∆PRD-13MYC::KanMX, sla1∆::NatMX, sla1W41AW108A::URA* |
|  |  |
| YSY3516 | *YSY3455* cross with *sla1W41AW108A::URA, sla1∆::NatMX, MYO5-GFP::HIS* |
|  |  |
| YSY3517 | *YSY3455* cross with *sla1W41AW108A::URA, sla1∆::NatMX, BBC1-GFP::HIS* |
|  |  |
| YSY3655 | *MATa ura3-52 leu2-3,112 his3Δ200 LAS17-end3C-GFP::HIS, ABP1-RFP::HygMX* |
|  |  |
| YSY3445 | *MATa ura3-52 leu2-3,112 his3Δ200 LAS17-GFP::HIS ABP1-RFP::HIS* |
|  |  |
| YSY3730 | *MATa ura3-52 leu2-3,112 his3Δ200 LAS17-end3C-GFP::HIS, pan1∆PRD-13MYC::KanMX, sla1W41AW108A:URA, sla1∆::NatMX, ABP1-RFP::HygMX* |
|  |  |
| YSY3654 | *MATa ura3-52 leu2-3,112 his3Δ200 LAS17-end3C-GFP::HIS, pan1∆PRD-13MYC::KanMX, sla1∆::cgLEU, ABP1-RFP::HygMX* |
|  |  |
| YSY3778 | *MATa ura3-52 leu2-3,112 his3Δ200 LAS17-end3C-GFP::HIS, sla1W41AW108:;URA, sla1∆::NatMX, pan1 ∆PRD-mCherry::KanMX,* |
|  |  |
| YSY3526 | *MATa ura3-52 leu2-3,112 his3Δ200 sla1W41AW108A::URA, sla1∆::NatMX, Pan1∆PRD-13MYC::KanMX, Abp1-RFP::HygMX* |
|  |  |
| YSY3737 | *MATa ura3-52 leu2-3,112 his3Δ200 VRP1-end3C-GFP::HIS, pan1∆PRD-13MYC::KanMX, sla1W41AW108A::URA, sla1∆::LEU, ABP1-RFP::HygMX* |
|  |  |
| YSY3742 | *MATa ura3-52 leu2-3,112 his3Δ200 BZZ1-end3C-GFP::HIS, pan ∆PRD-13MYC::KanMX, sla1W41AW108A::URA, sla1∆::LEU, ABP1-RFP::HygMX* |
|  |  |
| YSY3763 | *MATa ura3-52 leu2-3,112 his3Δ200 MYO5-end3C-GFP::HIS, ABP1-RFP::HygMX, sla1W41AW108A::URA, sla1∆::LEU, pan1∆PRD-13MYC::KanMX,* |
|  |  |
| YSY3770 | *MATa ura3-52 leu2-3,112 his3Δ200 RVS167-end3C-GFP::HIS, sla1W41AW108A::URA, sla1∆::NatMX, pan1 ∆PRD-13MYC::KanMX, ABP1-RFP::HygMX* |
|  |  |
| YSY3802 | *MATa ura3-52 leu2-3,112 his3Δ200 LSB4-end3C-GFP::HIS, sla1W41AW108A::URA, sla1∆::NatMX, pan1∆PRD-13MYC::KanMX* |
|  |  |
| YSY3681 | *MATa ura3-52 leu2-3,112 his3Δ200 LAS17-end3C-GFP::HIS, ABP1-RFP::HygMX, myo3∆::cgLEU,myo5CA∆-GFP::KanMX,* |
|  |  |
| YSY3823 | *MATa ura3-52 leu2-3,112 his3Δ200 myo5CA∆-GFP::KanMX, myo3∆::cgLEU, las17CA∆-GFP::HIS, ABP1RFP::HIS* |
|  |  |
| YSY3704 | *MATa ura3-52 leu2-3,112 his3Δ200 RVS167-end3C-GFP::HIS, ABP1-RFP::HygMX* |
|  |  |
| YSY3705 | *MATa ura3-52 leu2-3,112 his3Δ200 BZZ1-end3C-GFP::HIS, ABP1-RFP::HygMX* |
|  |  |
| YSY3706 | *MATa ura3-52 leu2-3,112 his3Δ200 MYO5-end3C-GFP::HIS, ABP1-RFP::HygMX, myo3∆::cgLEU* |
|  |  |
| YSY3808 | *MATa ura3-52 leu2-3,112 his3Δ200 LSB4-end3C-GFP::HIS, ABP1-RFP::HygMX* |
|  |  |
| YSY3661 | *MATa ura3-52 leu2-3,112 his3Δ200 VRP1-end3C-GFP::HIS, ABP1-RFP::HygMX* |
|  |  |
| YSY3932 | *MATa ura3-52 leu2-3,112 his3Δ200 EDE1-GFP::HIS, ABP1-RFP::HygMX, sla1W41AW108A::URA, sla1∆::NatMX, pan1 ∆PRD-13MYC::KanMX,* |
|  |  |
| YSY3786 | *MATa ura3-52 leu2-3,112 his3Δ200 VRP1-GFP::His, ABP1-RFP::HygMX,* |
|  |  |
| YSY3857 | *MATa ura3-52 leu2-3,112 his3Δ200 VRP1-end3C-GFP::HIS, sla1W41AW108A::URA, sla1∆::cgLEU, pan1 ∆PRD-13MYC::KanMX, LAS17-TagRFP-T::URA* |
|  |  |
| YSY3930 | *MATa ura3-52 leu2-3,112 his3Δ200 VRP1-end3C-GFP::HIS, sla1W41AW108A::URA, sla1∆::cgLEU, pan1 ∆PRD-13MYC::KanMX, MYO5-TagRFP-T::URA* |
|  |  |
| YSY4018 | *MATa ura3-52 leu2-3,112 his3Δ200 LAS17-GFP::HIS, MYO5-TagRFP-T::URA* |
|  |  |
| YSY4021 | *MATa ura3-52 leu2-3,112 his3Δ200 VRP1-GFP::HIS, MYO5-mCherry::KanMX* |
|  |  |
| YSY3785 | *MATa ura3-52 leu2-3,112 his3Δ200 MYO5-GFP::HIS, ABP1-RFP::HygMX* |
|  |  |
| YSY4026 | *MATa ura3-52 leu2-3,112 his3Δ200 vrp1∆::cgLEU, LAS17-end3 C-GFP::HIS, sla1W41AW108A::URA, sla1∆::NatMX, pan1 ∆PRD-13MYC::KanMX, ABP1-RFP::HygMX* |
|  |  |
| YSY4047 | *MATa ura3-52 leu2-3,112 his3Δ200 VRP1-end3C-GFP::HIS, sla1W41AW108A::URA, sla1∆::cgLEU, pan1 ∆PRD-13MYC::KanMX, ABP1-RFP::HygMX, las17∆::NatR* |
|  |  |
| YSY4054 | *MATa ura3-52 leu2-3,112 his3Δ200 MYO5-end3C-GFP::HIS, myo3∆::cgLEU, LAS17-TagRFP-T::URA* |
|  |  |
| YSY4049 | *MATa ura3-52 leu2-3,112 his3Δ200 MYO5-end3C-GFP::HIS, myo3∆::cgLEU, VRP1-mCherry::KanMX* |
|  |  |
| YSY4066 | *MATa ura3-52 leu2-3,112 his3Δ200 myo3∆::cgLEU, las17CA∆::HIS, MYO5-end3C-GFP::HIS* |
|  |  |
| YSY4071 | *MATa ura3-52 leu2-3,112 his3Δ200 myo3∆::cgLEU, las17CA∆::HIS, myo5-CA∆-GFP::HIS* |
|  |  |
